# Fast selection of miRNA candidates based on large-scale pre-computed MFE sets of randomized sequences

**DOI:** 10.1186/1756-0500-7-34

**Published:** 2014-01-13

**Authors:** Sven Warris, Sander Boymans, Iwe Muiser, Michiel Noback, Wim Krijnen, Jan-Peter Nap

**Affiliations:** 1 Expertise Centre ALIFE, Institute for Life Science & Technology, Hanze University of Applied Sciences, Groningen, The Netherlands; 2 Hubrecht Institute, Utrecht, The Netherlands

**Keywords:** MicroRNA, Minimal free energy, Grid computing, Epstein-Barr virus

## Abstract

**Background:**

Small RNAs are important regulators of genome function, yet their prediction in genomes is still a major computational challenge. Statistical analyses of pre-miRNA sequences indicated that their 2D structure tends to have a minimal free energy (MFE) significantly lower than MFE values of equivalently randomized sequences with the same nucleotide composition, in contrast to other classes of non-coding RNA. The computation of many MFEs is, however, too intensive to allow for genome-wide screenings.

**Results:**

Using a local grid infrastructure, MFE distributions of random sequences were pre-calculated on a large scale. These distributions follow a normal distribution and can be used to determine the MFE distribution for any given sequence composition by interpolation. It allows on-the-fly calculation of the normal distribution for any candidate sequence composition.

**Conclusion:**

The speedup achieved makes genome-wide screening with this characteristic of a pre-miRNA sequence practical. Although this particular property alone will not be able to distinguish miRNAs from other sequences sufficiently discriminative, the MFE-based P-value should be added to the parameters of choice to be included in the selection of potential miRNA candidates for experimental verification.

## Background

Small RNAs are important molecules in the regulation of gene expression. Several classes of distinct small RNA molecules play vital roles in development, health and disease, as well as in many other biological pathways [1-4]. A particular class of regulatory small RNAs are the microRNA (miRNA) molecules. A miRNA is a ~20-23 nucleotide (nt) short, non-protein coding RNA. Together with several protein components, miRNAs reduce the amount of a target mRNA by physical interaction to notably the 3′-untranslated region (3′-UTR) of the mRNA, resulting in either degradation of that mRNA, or arrest of translation [4-6]. In rare cases, miRNAs can also upregulate expression [4,7]. Well over 25 thousand miRNAs (miRBase Release 19; August 2012 [8]) have now been identified in many different species [9,10].

The overall biogenesis of miRNAs is well established [4,11], although details are still being discovered. In all cases except for intronic miRNAs [12], the miRNA is synthesized as a longer primary transcript known as primary miRNA (pri-miRNA), that is processed in the nucleus by the RNAse Drosha in animals and Dicer-like 1 in plants, to generate a precursor miRNA (pre-miRNA) of about 80–100 nt in animals, 60–300 nt in plants or 60–120 nt in (animal) viruses. The pre-miRNA sequence has degenerated palindromic sequence with the characteristic secondary structure of a stem-loop hairpin. The final verdict on the total number of miRNAs in a given genome is not out yet. The total count in Release 19 of miRBase is 25,141 for all organisms and many more miRNAs are described in the primary literature. Whereas the search for miRNAs in model genomes such as human or Arabidopsis will approach saturation, identification of the full miRNA complement in other genomes is still a challenge.

As mature miRNAs are only ~22 nt in length, straight-forward alignment-based heuristic methods such as BLAST are less suitable for identifying miRNAs and their targets in a given genome or transcriptome [5,13]. The identification of miRNAs and their targets is therefore a challenge for computational pattern recognition [11,14]. Computational methods for miRNA identification focus on the typical extended stem-loop hairpin structure of the pre-miRNA, which is characterized by helical base pairing with a few internal bulges in the stem. To identify the stem-loop miRNA precursor structure from a given sequence, RNA folding programs are used, such as mfold [15], its update UNAfold [16], or RNAfold (also known as the Vienna package [17,18], to establish the minimal free energy (MFE) of the stem-loop structure.

Increasingly sophisticated computational approaches have been proposed for the identification of pre-miRNAs, the mature miRNA sequence and its presumed target(s) [19-21], many of which are available online [22]. Many approaches are based on supposed or derived characteristics of miRNA sequences or combinations thereof [23-26]. Although all miRNAs are thought to have such properties in common, not a single property individually seems able to distinguish miRNAs sufficiently accurately from other RNA molecules with sufficient accuracy [27]. Several approaches therefore include evolutionary conservation of miRNA sequences between different species [1,28]. In these evolution-based strategies, species-specific and non -or less- conserved pre-miRNA molecules are likely to escape identification. Overall, methods available tend to show relatively high rates of false positives [22] and are possibly hampered by the use of inappropriate controls [29]. They generally result in lists too long to be feasible for experimental validation.

We here revisit a selective criterion proposed earlier, but largely unexplored because of computational costs. Statistical analyses of pre-miRNA hairpins indicated that such hairpins tend to have MFE values which are significantly lower than the MFE values based on randomized sequences with the same length and nucleotide composition, in contrast to other classes of RNA, such as transfer RNA, ribosomal RNA and messenger RNA [30-32]. In MFE analysis, the sequence composition of each candidate sequence is randomized and the MFE value based on the candidate is compared to the MFE distribution based on the randomized sequences. These data are used to calculate the probability that the MFE of the candidate is sufficiently small compared to randomized sequences [30]. This probability is here coined the empirical P-value (P_E_). This P_E_ establishes a useful discriminating criterion for pre-miRNA identification. It is implemented in the MiPred prediction tool [33], that helps to decide for a single sequence whether it is a pre-miRNA hairpin. However, the computation of large numbers of MFE values per candidate sequence to be able to calculate P_E_ is computationally demanding, which precludes application to genome-wide analyses. Solutions proposed in the literature are a probabilistic implementation of the MFE computation [34] or asymptotic *Z*-scores of the MFE distribution based on precomputed tables [35]. We here present a novel approach that requires the computation of only the MFE based on the candidate sequence. This approach enables the routine evaluation of potential miRNA structures on a genome-wide scale that could be integrated as part of an existing approach for processing potential miRNA sequences [20,36].

## Methods

### Data

The miRNA data set was downloaded from the miRbase repository [8-10] (releases 9.2 and 15), consisting of 4,584 and 15,172 pre-miRNA sequences respectively. The genomic sequence of the Epstein Barr virus type 1 [37] was downloaded from Genbank NCBI [gi|82503188|ref|NC_007605.1]. The test set with 250,000 random sequences was generated with a small C program.

### Hardware

Computations were performed on a 200 + −node Debian Linux-based network. A dedicated server is running Network File System (NFS)-based software for file management and Condor software ([38]; version 7.6.1) for grid management [39].

### RNA folding software

The minimal free energy of a sequence was computed with a local implementation of the Hybrid software (version 2.5) of the UNAFold software package [16,40]. UNAFold extends and replaces the earlier mfold application [15]. The software was adjusted to enhance the performance about three-fold by optimizing computation-intensive computational steps without changing the underlying algorithm. All RNA molecules were folded as single strands at 30°C, a sodium concentration of 1.0 M and the option –E (energy only, no plots). In case of sequences that are not able to fold properly, the Hybrid software assigns an MFE of + ∞.

### Randomization and visualization

Distribution fitting, P-values and other statistics were computed with the software suite R (version 2.7.1.) [41]. To randomize sequences while maintaining the nucleotide composition, the Fisher-Yates shuffling procedure for selection without replacement was implemented in C, with appropriate unbiased randomization [42]. For any candidate sequence, the empirical P-value P_E_ was computed as P_E_ = X/(N + 1) [30], where X is the number of sequences with an MFE lower than or equal to the MFE based on the candidate sequence and N is the number of randomized sequences considered. In this study, N is taken as 1,000, in correspondence with an earlier study [30]. As a consequence, the lower bound of P_E_ is zero (for X = 0) and the next lowest value is 0.000999 (for X = 1). There are no additional assumptions necessary with respect to the shape of the distribution of the MFE values [30]. The computed MFE values based on the randomized sequences were transformed into a normal (Gaussian) distribution defined by the mean and standard deviation of the MFE values. The normal distribution-derived P_N_ of the MFE based on the given candidate sequence is being computed using the mean and standard deviation of that distribution. Results were visualized with R and MatLab (release 13).

### Multidimensional interpolation

A database of entry RNA sequences, with a length of 50 to 300 nt and a step size of 5 nt, was generated by computer. This range covers the length of most known pre-miRNAs, except for some plant miRNAs [43]. For each sequence length, the nucleotide composition of the sequence was varied in such a way that each of the four nucleotides occurs at least once (for sequences < 100 nt) or at least at 1% (for sequences > 100 nt). Per sequence length, individual sequences were generated with a step size for an individual nucleotide of 2%, except in the range from 20-70% for an individual nucleotide where a step size of 1% was used. The procedure in numbers is as follows: for a population of sequences with a length of 50nt, the first nucleotide composition consists of 1% A, 1% U, 1% C and 97% G. In the next step the composition is 2% A, 1% U, 1% C and 96% G and so on. Then the length is increased to 55 nt and the procedure is repeated for the nucleotide composition, etc. This procedure generated a set of 1.4 × 10^6^ entry sequences. For each of the individual entry sequences, a sequence set of thousand randomized shuffles was generated by Fisher-Yates randomization [42]. This procedure represents a selection without replacement, therefore maintains the nucleotide composition (mononucleotide shuffling). Sequence sets in which one or more shuffled sequences had an MFE of + ∞ were discarded and only the sequence sets with 1,000 MFE values were considered to maintain statistical validity. This way, a total of 1.05 × 10^6^ sequence sets were generated. For each population, the mean MFE and standard deviation were computed and stored in a MySQL database together with the sequence composition in absolute nucleotide counts. To calculate the mean and standard deviation for any candidate sequence, an interpolation algorithm was implemented in C++ using sparse matrix data management for optimal memory use [44]. A sparse matrix contains only the values of interest and all zero or unknown values are not stored. The resulting data structure contains only the nucleotide composition analysed and not all possible compositions, therefore the data can be stored in memory and searched efficiently. The Hybrid software used for RNA folding [16] was integrated within this application to enhance performance.

### Sliding window analysis

To analyse whole genomes for the presence of potential pre-miRNA candidates using the pre-computed MFE data outlined above, a sliding window approach was implemented in C++. The smallest window length was set at 50 nt, incremented with a step size of 10 nt to a maximum of 300 nt. For each window length, the step size for sliding was set at 10% of the window length. For each window, the MFE was computed and the nucleotide composition of the sequence was determined. Based on the sequence composition, the appropriate mean and standard deviation were estimated by interpolation (see Results) using the data search space generated. The normal distribution function was used to calculate P_N_ of the MFE of the window.

## Results and discussion

In the identification of potential pre-miRNA candidates in genomic sequences, the MFE based on the sequence relative to the distribution of random sequences with the same nucleotide composition is a potentially valuable criterion. However, the estimation of the empirical P_E_ as parameter for the distance between the MFE based on a candidate sequence and the MFEs of randomized sequences is computationally intensive. It requires the computation of the MFE for all randomized sequences. To use the MFE distribution as criterion more comfortably, computations should be considerably faster. We here show the feasibility of the use of the normal distribution for the computation of P_N_ as approximation of P_E_ and for the interpolation of the distribution for any given sequence with the help of pre-computed MFE distributions of random sequences.

### Pre-computed MFE distributions of random sequences

A total of 1.4 × 10^6^ entry sequences covering the length classes representative for most known pre-miRNA (50 – 300 nt), were generated. Each entry sequence was shuffled 1,000 times and based on each of the generated sequences the MFE was calculated giving a total of 1,053,248 populations, each consisting of 1,000 random sequences. In 346,752 generated populations one or more random sequence could not fold properly. When the hybrid software is not able to give a stable structure, the random sequence is considered not to fold properly and is therefore not included because it skews the data. For a single sequence on a standard desktop PC, the MFE computation by the Hybrid software requires approximately 0.2 sec CPU time. The 1.4 × 10^6^ × 1,000 computations would therefore have taken about 8.8 CPU years on a standard PC. Using idle CPU cycles on our grid, it took about 2 months grid time to complete all computations. All MFE values were computed for an annealing temperature of 30°C, but as MFE values and distributions change in a linear way with temperature (results not shown), the approach presented and data generated are, if so desired, suitable for, or comparable with, other folding temperatures.

**Figure 1:**
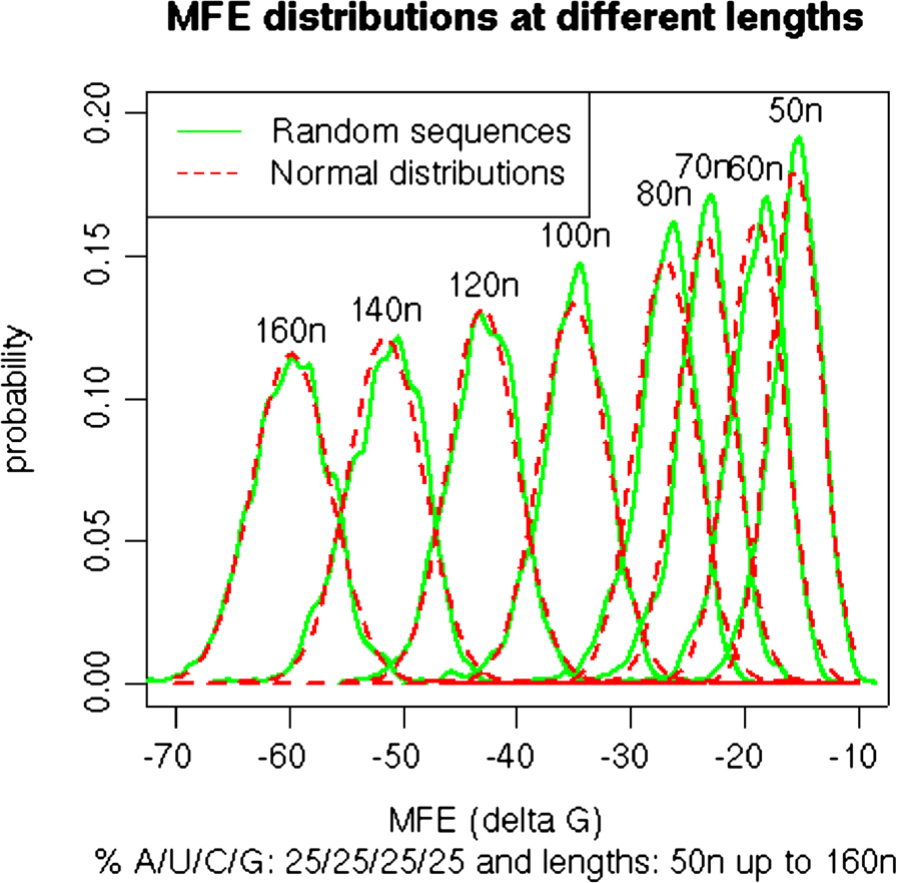
**Distribution of P**_**E **_**and P**_**N **_**of sequences of different lengths.** For a candidate sequence with the given length in nucleotides *n* (50 to 160) and a composition of 25% of each nucleotide (AUGC), the MFE of 1000 randomized sequences was calculated. The distribution was computed and plotted (green) using the distribution density function in R. The average mean and standard deviation of the resulting MFE sequence set was used to define the normal distribution function (red). The good correspondence between the two distributions shows that the normal distribution-based probability P_N_ is a good approximation for the empirical probability P_E_.

Randomized sequence sets can reasonably be considered to reflect a normal distribution. The examples for sets with 25% nucleotide composition are shown in Figure 1. Other compositions give similar results (data not shown). Such a normal distribution was demonstrated earlier for randomized sequences [45], although the distribution may not be an exact Gaussian distribution [34]. The MFE data of random sequences are therefore suitable for deriving the normal estimate P_N_ of P_E_, based on mean, standard deviation and the normal distribution function. This way, P_N_ is equivalent to the *Z*-score of the MFE, defined as the number of standard deviations by which the MFE based on a candidate sequence deviates from the mean MFE of the set of shuffled sequences [31,45].

**Figure 2:**
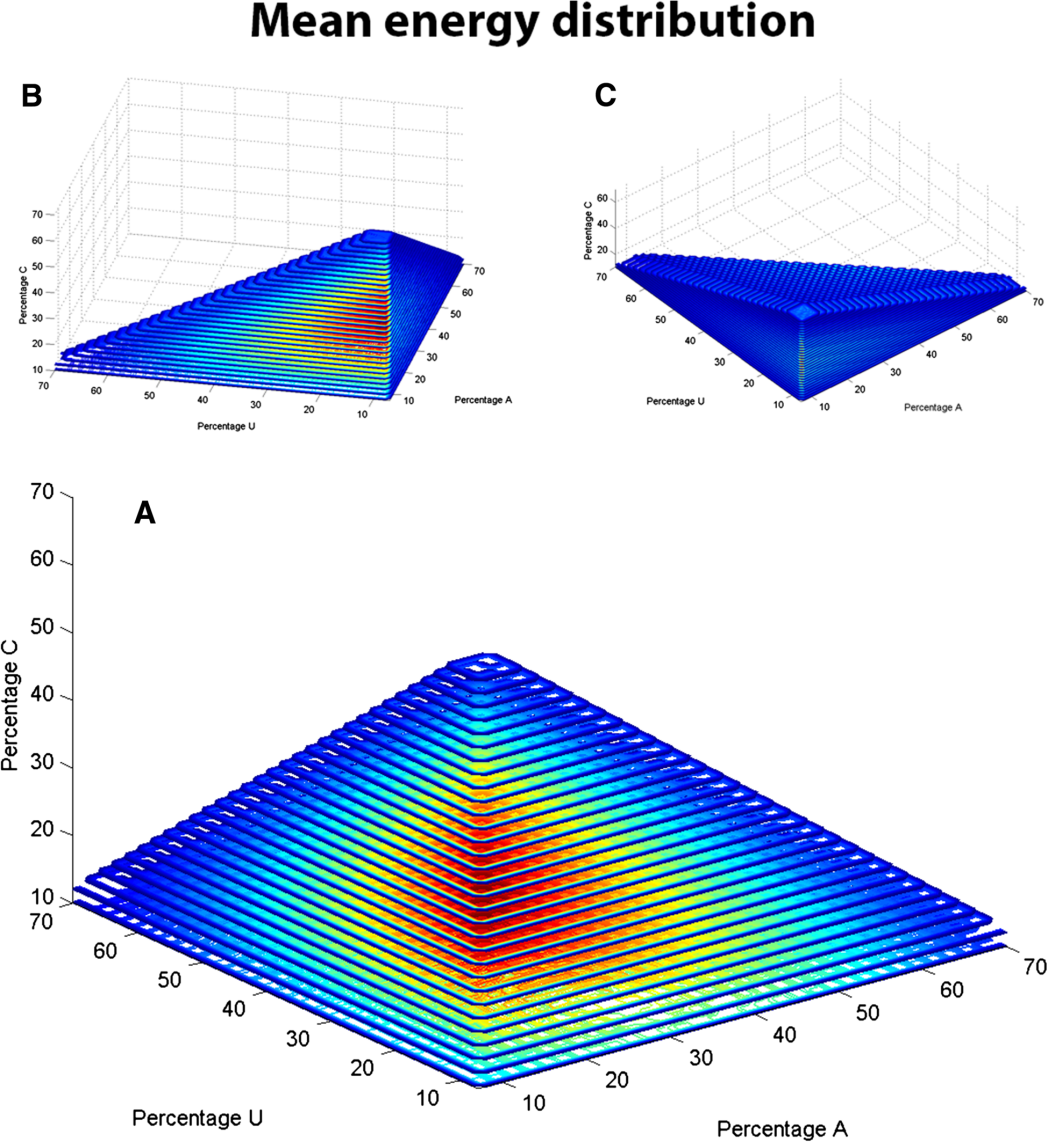
**Mean MFE distribution of sequences 100 nt in length. (A)** The mean MFE of 1000 sequences with the indicated composition is plotted in a 3D contour plot (Matlab) with the percentage of three nucleotides in the sequence specified on the three axes. The false color scale indicates a relative measure of the mean MFE: red a relatively low MFE value with a ΔG (Gibbs free energy change) ≤ −80 kcal/mol, yellow an intermediate MFE value (−80 kcal/mol < ΔG ≤ −40 kcal/mol) and blue a relatively high MFE value (ΔG > −40 kcal/mol). **(B,C)** The same distribution as in (A) is shown at two different angles to help interpretation and to prevent optical illusions.

For each sequence set, the mean and standard deviation was stored in a database together with the sequence composition. An example of the distribution of the mean MFE value of all sequence sets of 100 nt in length with different sequence compositions is shown in Figure 2. The 3D contour plot shows that the sets of sequences with high percentages of C and G nucleotides have low mean MFE values, which reflects the higher energy in C-G pairing. RNA molecules with an abundance of for example A and G are much less likely to form a stable structure and the set of 1,000 random sequences will therefore have a high mean MFE. The plot shows that the mean MFE values decrease in an almost linear fashion from the low values for sequences with high C and G compositions to the outer edges.

### Multidimensional interpolation for candidate sequences

For the on-the-fly computation of the MFE distribution based on a given candidate sequence, the pre-computed data are used for multidimensional interpolation. For each candidate sequence, the composition of the sequence is determined by counting nucleotides. Sequence compositions with a squared Euclidean distance up to 5 in the surrounding search space are identified: the length of the sequence is therefore not taken into account. This value was selected on the basis of the analysis of known miRNAs. These analyses showed this threshold gives the smallest difference in P-value (data not shown) when comparing the P_N_ to the P_E_ of mirbase entries. For sequence compositions that have no points within this distance no prediction can be made and a P_N_ of 1.0 is given. From the selected near-by sequences, the mean and standard deviations are retrieved from the database. For the candidate sequence, both mean and standard deviation of the MFE distribution are determined by interpolation using the data from the nearby sequence compositions, weighted based on their Euclidean distance to the candidate sequence. The sum of the weighted values gives the estimated mean and standard deviation of the MFE distribution based on the randomized sequences from the candidate sequence. Mathematically, the formulae to derive the estimated average *μ*_*e*_ are expressed as:

$$\begin{array}{l} {\bar{\mu }}_{e}=\frac{\sum_{i=0}^{N-1}\omega_{i}\mu_{i}}{\sum_{i=0}^{N-1}\omega_{i}}\;\mathrm{with}\;\omega_{i}=\frac{d_{t}-d_{i}}{d_{t}},\\ d_{i}=\sqrt{\sum_{i=0}^{3}{\left(x_{i}-y_{i}\right)}^{2}}\;\mathrm{and}\;d_{t}=\sum_{i=0}^{N-1}d_{i} \end{array}$$

where *w*_*i*_ is the weight per data point based on Euclidean distance, *d*_*i*_ is the distance per point, for which x_i_ and y_i_ are the nucleotide counts of the sequence in the search space and *d*_*t*_ is the total distance over N points. N varies per candidate sequence. Even in the case were the distance to a point is zero (d_i_ = 0), more points are used to estimate the population average. Testing on sequences with the same compositions during computations would slow the software down and this situation is unlikely therefore it was not included in the software.

The use of P_N_ as normal approximation of P_E_ was evaluated by comparing both probabilities for different sequences. In Figure 1, the comparison between many P_E_ (green) and P_N_ (red) is shown for a range of sequences with different lengths but the same nucleotide compositions. Other compositions give similar results (data not shown). The excellent goodness-of-fit demonstrates the suitability of the normal approximation P_N_ as criterion for the evaluation of pre-miRNA candidate sequences.

The estimation of the standard deviation of the MFE distribution based on the candidate sequence is based on the approach for estimating the mean as described in the previous section. The mean and standard deviation uniquely define the normal distribution function of the candidate sequence. With the two values, P_N_ is computed as the normal probability of MFE values smaller than the MFE based on the candidate sequence. This way, for each candidate sequence, only the MFE of the structure based on this sequence needs to be computed, speeding up computations approximately a thousand-fold when thousand shuffled sequences are used. As the calculations of the estimated mean, standard deviation and the P-value based on this normal distribution take time as well, the software is at least several hundred times faster for short sequences and faster for long candidates: the Hybrid software used is slower for longer sequences, as there are more secondary structures. Calculating the structures of 1000 candidates takes therefore considerably more time than the estimation process. Based on the running time of an example run it would take over 260 seconds to calculate 1000 MFE values. The calculation of P_N_ takes approximate a second, including the calculation of the MFE.

**Figure 3:**
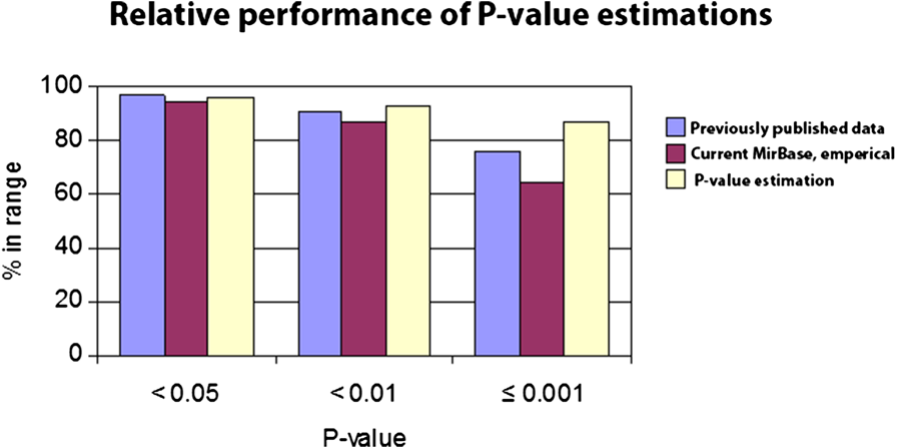
**Relative performance of MFE-based P-value estimations.** The percentage of pre-miRNAs with a P-value smaller than indicated is plotted for data previously published [30], newly computed values from release 9.2 of MirBase based on the same method and computed based on the interpolation method developed here. The previously published percentages based on P_E_ were 97%, 91% and 76%, respectively, whereas based on the release 9.2 it is 95%, 87% and 65%, and P_N_ 96%, 93% and 87%, respectively.

We evaluated the performance of P_N_ compared to P_E_ for selection with respect to the entries in the MiRBase registry. In Figure 3, the distribution of P_E_ over the pre-miRNA molecules as published previously [30] is compared with the P_E_ computed of the current pre-miRNAs from miRbase with the same method [30] and the P_N_ as estimated by interpolation. It shows that the interpolation approach performs well. The difference between P_E_ and P_N_ reflects that the P_N_ distribution is continuous, whereas with 1,000 randomizations, the P_E_ distribution is discrete with a step size of 1/1001 = 0.000999. Although also P_N_ is estimated on the basis of 1,000 randomizations, the continuity of the distribution allows more strict settings for P_N_ and allows a more sensitive ranking in the lower P-value ranges. In Table 1, the percentages of pre-miRNAs that conform to given settings of the P-value are shown. With a threshold of P_N_ = 1×10^-4^, about 66.8% of all known pre-miRNAs are characterized by an MFE value well outside the distribution of MFE values of randomized sequences. Only 1% could not be estimated: the sequence was either too long or had no populations in the data set within the given distance.

**Table 1:** Percentage of sequences with low P-values

**Data set**	**Total # sequences analyzed**	**% sequences with an error/not found**	**% sequences with P <0.05**	**% sequences with P <0.01**	**% sequences with P < =0.001**	**% sequences with P < =0.0001**
Random	250000	21.3	3.0	1.5	0.8	0.5
MirBase	15172	1.0	89.9	84.4	75.9	66.8
EBV known miRNAs	25	0.0	96.0	92.0	88.0	76.0
EBV genome	566988	0.1	19.1	10.7	5.7	3.6

This table shows the percentage of sequences below the given P-value in four different data sets. A data set of random sequences shows a very low percentage with a P-value < = 0.001. A high percentage of known miRNAs sequences is within the same range.

To verify the performance of the applications on random sequences, a test set of 250,000 sequences was generated; all of different lengths and composition. The MFE and P_N_ were calculated for each of these sequences. The calculations were finished in 20 minutes. In Table 1 the results are shown. Of these random sequences, 4% had no stable structure and hence no MFE and 17% had no populations in the data set within the given distance. Of the remaining sequences only 3% had a P_N_ < 0.05.

### Shuffling inconsistencies

**Figure 4:**
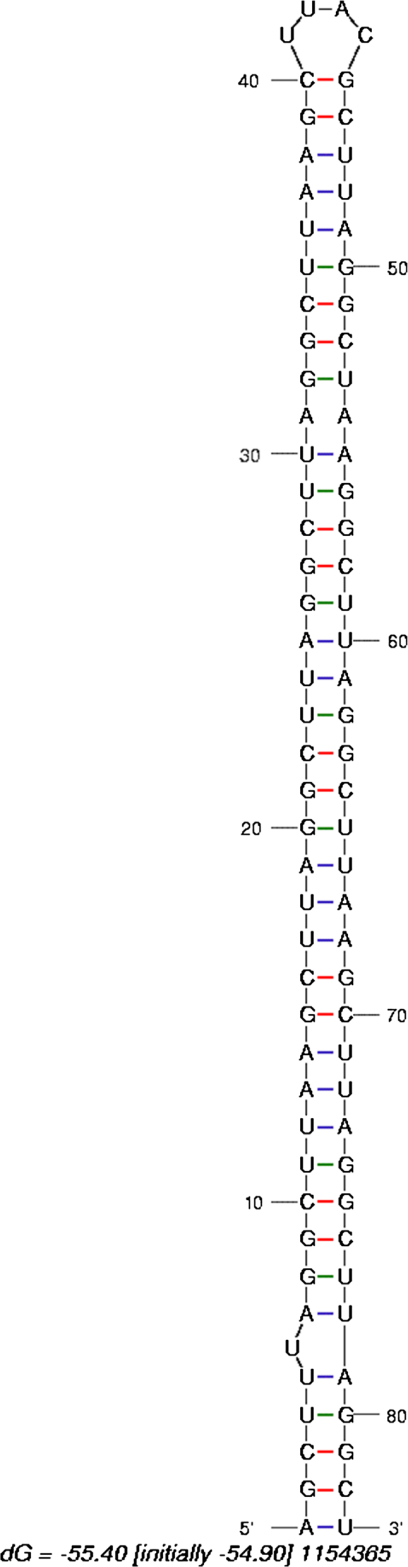
**Hairpin with internal repeat structure.** Example of an RNA sequence with a large difference in P_N_ versus P_E_, depending on the method of randomization (see text for details).

In the analyses above, a mononucleotide randomization method (Fisher-Yates algorithm) was used [42], whereas in the literature a dinucleotide randomization method was recommended and used [30,31,46]. In genome-wide analyses of candidate sequences based on P_N_, we observed that several candidate sequences behaved oddly: whereas dinucleotide shuffling yielded P_N_ = 0,98, reflecting not a likely candidate, mononucleotide shuffling as performed here resulted in a P_N_ < < 0.001, indicating a possible candidate. An example of such a sequence is given in Figure 4, which shows a predicted hairpin structure for this sequence. To prevent such sequences interfering with the analysis of the distribution, the mononucleotide randomization method was used.

### Added value for miRNA prediction

**Figure 5:**
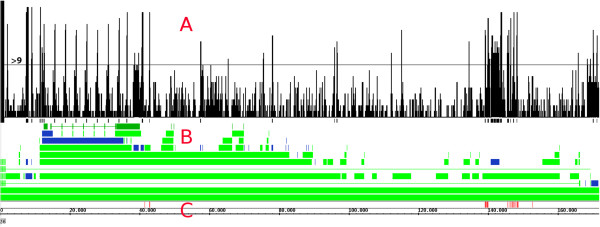
**Identification of potential pre-miRNA candidates in the Epstein-Barr virus genome sequence.** The genomic sequence is shown on the x-axis. The upper track (red A) shows the amount of windows covering the particular region that have a P_N_ < = 0.001. A distinct peak gives a region of interest for a candidate miRNA. By discarding peaks within a repeat region (here shown in blue) and selecting peaks at or above 9 hits, 18 new regions of interest are found (plus strand). Also, 12 known miRNA are found. The green bars indicated by the red B show the EBV genome annotation (gi|82503188|ref|NC_007605.1). The lower part of the graph (red C) shows the EBV genome locations with the red bars indicating locations of the known miRNAs.

Having validated the P_N_ interpolation method with known miRNAs, we now show the added value for whole genome screening. We have evaluated a small viral genome for putative pre-miRNAs regions. According to miRBase (both in release 15 and 19), this viral genome has 25 known pre-miRNA sequences. Of the 25 known Epstein-Barr virus (EBV) miRNAs, 24 have a P_N_ < 0.05 with 22 having a P_N_ < = 0.001 (Table 1). Both strands of the dsDNA genome sequence of the human Epstein-Barr virus type 1 [37] were converted into RNA and investigated for potential pre-miRNA sequence. For each of the total 566,988 windows of length 50–230 nt, the MFE and the P_N_ were computed. In contrast to the test set with random sequences, in EBV only 0.05% of the windows could not be estimated due to no sequence compositions within the given distance or because the sequence did not have a Stable 2D structure. This shows that the application performs very well for viral genomic DNA. The percentage of windows with a P_N_ < =0.001 is higher than in the test set with random sequences (Table 1). There is also the effect of overlapping windows: a pre-miRNA of length 150 will be found in several windows of length 200. Many of these candidate windows are in repeat regions (Figure 5). These windows can be discarded as not being viable locations for miRNAs: there are no known miRNAs within the EBV repeat regions. Although current research shows that in some organisms miRNAs can be found in repeat regions [47], we suggest inspection of other regions first to limit the number of relevant candidate regions.

To visualize regions of interest, the windows with a P_N_ < = 0.001 are placed in a separate data set and marked with a value of one. The windows of different lengths are then combined in the Integrated Genome Browser [48] (Figure 5). With 19 different window lengths, the maximum of the resulting graph is 19. This indicates that all windows covering this location have a P_N_ < =0.001. The peaks in the graph show regions of interest which require further research. The graph shows 18 regions-of-interest in the plus strand where 9 or more windows have P_N_ < = 0.001. Using these selection criteria, the regions of 12 known miRNAs are found (Figure 5). Using less than 9 windows will give more regions-of-interest and will also show more known miRNA regions, but will introduce more false positives as well. The analysis of the EBV genome shows that a (small) whole genome screening using P_N_-estimation results in a limited number of regions-of-interest for further investigation.

## Conclusion

Previous research has indicated that the MFE based on a miRNA sequence is significantly lower than the MFE based on shuffled sequences with the same composition, in contrast to the MFE of other non-coding RNAs [30,31,49]. As the computation of an MFE is demanding, this characteristic of miRNAs precludes genome screening of sequences for their MFE distribution. With thousand randomizations per candidate sequence, the genome-wide screening of a million (10^6^) candidates would require a billion (10^9^) computations. These would take well over six year to finish on a current standard desktop computer.

We have presented a method to speed up analyses of the MFE distribution considerably, based on the normal approximation of pre-calculated MFE distributions based on random sequences, combined with a fast implementation of a multidimensional interpolation of distributions in sequence space. The data cover the search space for all RNA molecules with a length from 50 to 300 nt, in total roughly equaling the sum over 4^i^ for i = 50….300 ≈ 5.5.10^180^ sequences. With three data points per sequence (mean, standard deviation and composition), this would generate an immense database, whereas the resulting data space here established is based on 1.1 × 10^6^ sequences and takes about 30 Mb. The latter is easily handled by standard amounts of RAM. The results show that although the newer miRNAs added to miRBase since 2006 seems to comply somewhat less with this criterion than the miRNAs analyzed before [50], the new approach developed here performs well on known pre-miRNAs (Figure 3).

Sequence sets of 1,000 with at least one non-folding member were discarded. Yet, it could be argued that higher accuracy would be gained with more sets. The data is well distributed over the sequence data space (Figure 2). The interpolation of MFE distributions is based on a threshold of the Euclidian distance of the surrounding data points. This implies that for different candidate sequences different amounts of pre-computed data are used to estimate the MFE distribution. This prevents interpolation issues at the boundaries of the data space where less points are available. The data reduction and interpolation results in considerably faster computation of the likelihood that the MFE of the sequence is markedly lower than equivalent randomized sequences. This obviates the need for on-the-fly computation of the MFE values based on the randomized sequences.

The particular type and number of randomizations is an issue. Whereas it was thought to be important to maintain not only the mononucleotide compositions, but also the dinucleotide distribution [46], the results shown for the behavior of miRNAs in either way of shuffling [30,51] indicate no relevant difference, or even a slightly better performance of mononucleotide shuffling. These findings indicate that for miRNA prediction dinucleotide shuffling is not more optimal than mononucleotide shuffling. This is in agreement with the demonstration that all base pairings in an RNA molecule should be taken into account [52]. There is, in addition, uncertainty over the quality of the dinucleotide shuffling algorithm [35,51]. As demonstrated here, particular sequences behave oddly with respect to dinucleotide shuffling (Figure 4) and may distort the distribution derived from the computed MFE values. Inspection of such sequences indicated that these sequences contain a particular combination of repeat units in such a way that the dinucleotide shuffling is not changing the sequence in terms of MFE distribution. As a result, the candidate MFE is part of the distribution of shuffled sequences. As Fisher-Yates shuffling is the most random, this would seem to be the better method. We have followed the earlier recommendation of performing at least 1,000 randomizations [30], whereas other investigations use 500 [31] or 10,000 [51]. As few as 100 randomizations were recommended as sufficient to establish a reasonable Gaussian distribution [45].

In view of the gain in computing speed accomplished with P_N_, it has become feasible to consider genome-wide screenings for pre-miRNA candidates based on P_N_*.* The analyses here presented for the relatively small Epstein Barr virus demonstrate that indeed such analysis is now within reach. For a human genome, however, the approach will still ask a considerable computational effort. Moreover, the MFE alone is not able to distinguish miRNAs from other sequences sufficiently discriminative: it has to be integrated with other parameters. The P_N_ approach presented here can therefore be better implemented as part of, or next to, other approaches [20,22]. Such approach would generate added value for such miRNA identification algorithms or pipelines. The application of this criterion will add to enhanced selectivity of miRNA discovery pipelines and help to limit the number of candidates for experimental validation and confirmation.

The advent of high throughput DNA sequencing technologies were shown to be particularly suitable for the analyses of the small RNA complement of RNA populations [4]. The identification of true miRNAs in such data sets is still a challenge to which the P_N_ analysis may contribute. The possibility of a one-time effort to pre-compute sequence parameters that will facilitate future analyses should be considered an approach that could generate considerable added value for larger grid environments in future bioinformatics.

## Competing interests

The authors declare that they have no competing interests.

## Authors’ contributions

SW invented the prediction method, performed most of the data analyses, including the EBV analyses, wrote the software and the manuscript. SB and IM created part of the data set and performed quality checks. MN and WK provided statistical and biological interpretations and analyses. JPN directed the research, provided additional biological interpretations and was involved in drafting the manuscript. All authors contributed to, read and approved the manuscript.
